# Prevalence of Influenza Viruses A and B, Adenovirus, Respiratory Syncytial Virus, and Human Metapneumonia Viruses among Children with Acute Respiratory Tract Infection

**DOI:** 10.1155/2024/7613948

**Published:** 2024-01-22

**Authors:** Rana Farzi, Neda Pirbonyeh, Mohammad Rahim Kadivar, Afagh Moattari

**Affiliations:** ^1^Department of Bacteriology and Virology, School of Medicine, Shiraz University of Medical Sciences, Shiraz, Iran; ^2^Burn and Wound Healing Research Center, Microbiology Department, Shiraz University of Medical Sciences, Shiraz, Iran; ^3^Department of Pediatrics, School of Medicine, Shiraz University of Medical Sciences, Shiraz, Iran; ^4^Professor Alborzi Clinical Microbiology Research Center, Namazi Hospital, Shiraz Medical University, Shiraz, Iran

## Abstract

**Background:**

Acute respiratory tract infection (ARTI) is a significant cause of morbidity and mortality among children worldwide. The majority of acute respiratory infections in children are caused by viruses, with respiratory syncytial virus (RSV) being the most frequently encountered. Other important viral pathogens include human metapneumovirus, human coronaviruses, adenovirus, and influenza. These infections can lead to complications such as bronchitis and pneumonia. So, this study aimed to evaluate the prevalence of influenza viruses A and B, adenovirus, respiratory syncytial virus (RSV), and human metapneumovirus (HMPV) in children with ARTI.

**Methods:**

The molecular diagnostic of polymerase chain reaction approach was used to detect influenza (A and B), metapneumovirus, respiratory syncytial virus (RSV), and adenovirus in respiratory samples of children with acute respiratory infection hospitalization in a teaching hospital of the Shiraz University of Medical Sciences in January 2016–March 2017.

**Results:**

Of the 340 patients examined, 208 (61.20%) were male and the median age was 3.13 ± 2.38 years. Respiratory viruses were found in 179 (52.64%) patients. The male-to-female ratio was 1.63 : 1 in patients who were viral positive. Detection rates for influenza A, adenovirus, influenza B, RSV, and HMPV were 28.23%, 24.70%, 8.52%, 3.23%, and 2.64%, respectively, and coinfections were detected in 24.02%. The most common combination of two-virus coinfections was IFVA/AdV, followed by IFVB/AdV, AdV, IFVB/IFVA, RSV/IFVA, HMPV/AdV, RSV/AdV, and HMPV/IFVA.

**Conclusion:**

The high prevalence of respiratory viruses in children hospitalized with ARTI suggests that viral infection may play a role in disease pathogenesis. This should be confirmed through the conduct of case-control studies and may inform the role of vaccination to prevent respiratory viral infections.

## 1. Introduction

**Figure 1:**
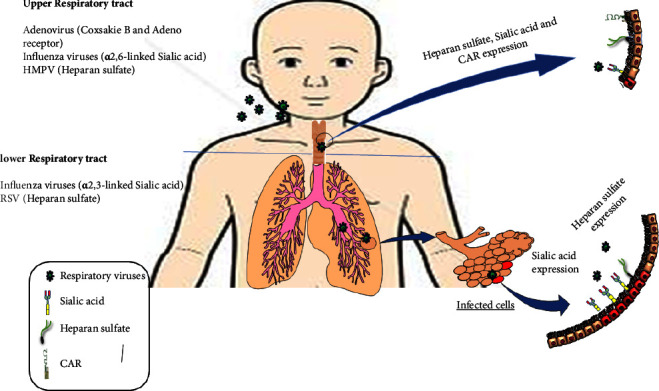
Occurrence of viral ART infection in children.

Community-acquired acute respiratory tract infection (ARTI) is a major cause of illness and fatality throughout the world [1, 2], Also, every year 4-5 million children in developing countries are hospitalized due to this infection [2, 3]. Older people, especially the elderly, young children, and immunocompromised people are more at risk of death [1]. ARTI is the most prevalent reason for referral to medical clinics and accounts for 70% of the respiratory diseases in young children and newborns under one year of age [4, 5]. These infections affect the upper and lower respiratory tracts from the pharynx to the alveoli, with a spectrum of disease ranging from mild cold to severe pneumonia illness [6]. ARTI includes rhinopharyngitis, pharyngitis, sinusitis, acute otitis media, epiglottitis, laryngitis, laryngotracheobronchitis, bronchitis, bronchiolitis, and exacerbation of asthma and pneumonia [7]. The circulation of these infections in the community is common and transmission occurs through the inhalation of aerosols and touching contaminated surfaces with self-inoculation onto mucosal surfaces [4]. These infections are easily transmitted and spread in the society rapidly and pose a huge economic burden on the society [6]. Among causative agents of the respiratory tract pathogens, viruses play a major role in the development of ARTI [4] and are responsible for 30%–40% of ARTIs [8]. Viruses commonly associated with ARTIs include respiratory syncytial virus (RSV), influenza virus A (IFVA), influenza virus B (IFVB), adenovirus (AdV), and human metapneumovirus (HMPV) [3, 9]. These viruses use specific receptor and generate infection in respiratory tract infection (Figure 1). Other viruses, including human parainfluenza viruses (HPIVs), rhinovirus (RV), and human coronavirus (HCov), are also associated with ARTI [10, 11]. Respiratory viral pathogens may cause ARTI as a single-pathogen infection or as coinfections with other viruses or bacteria. The most important bacterial agents involved in the respiratory infection are *Legionella pneumophila* (LP), *Chlamydophila pneumoniae* (CP), *Mycoplasma pneumoniae* (MP), and *Bordetella pertussis* (BP) and also Pneumococcus and Hemophilus influenzae have partial role in the respiratory infection [1, 12]. Several studies have suggested that coinfection may be associated with increased severity of ARTI [13–16]. Identification of the commonly occurring viruses informs prevention strategies, including vaccine production [4]. The aim of this study was to investigate the prevalence of IFVA, IFVB, AdV, RSV, and HMPV among children hospitalized with ARTI.

## 2. Methods

### 2.1. Study Population

This was a cross-sectional study of children under 14 years of age admitted with an LT-ARI diagnosis at hospitals affiliated with the Shiraz University of Medical Sciences in the period between January 2016 and March 2017. LT-ARI was defined as any acute lower respiratory tract infection of sufficient severity to warrant admission to the hospital. All types of LT-ARI were included, from bronchiolitis to pneumonia, with or without wheezing, fever, rhinorrhea, or respiratory distress.

### 2.2. Collection and Preparation of the Samples

Throat and nasopharyngeal swab samples were collected from the study participants. Samples were refrigerated at 2–8°C and transported on ice to the Virology Department of Shiraz University of Medical Science and stored at −80°C until analyzed.

### 2.3. Nucleic Acid Extraction and Virus Detection

Nucleic acid (DNA or RNA) extraction was performed by using the High Pure Viral Nucleic Acid kit (Roche, Mannheim, Germany) according to the manufacturer's instructions. For AdV DNA detection in the throat and nasopharyngeal samples, PCR was performed using Master Mix RED (Amplicon, A180306).

Detection of HMPV and RSV was performed using the one step RT-PCR kit (QIAGEN GmbH, Germany). IFVA and IFVB were detected using a one-step real-time (RT) PCR kit (QIAGEN GmbH, Germany), according to the manufacturer's instructions.

The primers and probe sequences used in this study are summarized in Table 1.

### 2.4. Statistical Analysis

Statistical analysis was performed using SPSS software, version 25. Chi-square or Fisher's exact tests were used for comparison of the proportions. The significance level was determined at *p* < 0.05.

### 2.5. Ethical Considerations

Ethical approval for this study was obtained from SUMS Medical Ethics (IR.SUMC.REC. 1396.S564). Written informed consent was obtained from all the study patients.

## 3. Result

Out of the 340 respiratory patients, 132 (38.80%) were female and 208 (61.20%) were male and the patients' median age was 3.13 ± 2.38 years and infection rates were higher in under three years and 3–5 age groups.

Investigation revealed that 179 (52.64%) patients with respiratory infection had at least one respiratory virus. Considering that the samples were taken from hospitalized patients with respiratory symptom in hospital wards during the peak of respiratory infections in autumn and winter, the high rates are not biased. Demographic data of all the children admitted with acute respiratory infections are shown in Table 2. The rate of viral respiratory infections was estimated at 68 (37.98%) for female and 111 (62.01) for male. The male-to-female ratio was 1.63 : 1 (111 : 68) in patients who were viral positive. The viral infection rates were more in <5 age. The respiratory virus-infected patients' median age was 3.30 + 2.46 years. In terms of disease symptoms, the most common symptoms in these patients include fever, cough, sore throat, muscle pain, wheezing, anorexia, runny nose, and headache.

Diarrhea and vomiting were also observed in some patients at a young age.

The frequency of each viral infection was IFVA: 96 (28.23%), AdV: 84 (24.70%), IFVB: 29 (8.52%), HMPV: 9 (2.64%), and RSV: 11 (3.23%) of the samples.

The most age group infected with the IFVA, IFVB, and AdV was the age group under 10 years old, and the most infected age group with RSV and HMPV was under 5 and under three years age group.

**Figure 2:**
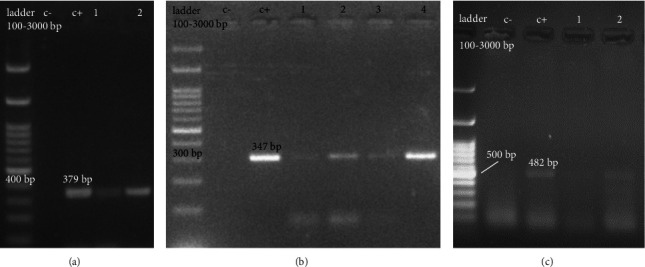
Detection of respiratory viruses. c−, negative control; c+, positive control. (a) Respiratory syncytial virus, (b) human metapneumonia viruses, and (c) adenovirus.

The rate of monovirus infection in patients with viral respiratory infections was 136/170 (80.00%). This was despite the fact that some children were infected with more than one virus (coinfection), and its frequency was 43/170 (25.29%) (Table 3). Agarose gel electrophoresis image of PCR of RSV (A), HMPV(B), AdV(c) is shown in Figures 2(a)–2(c).

Multiple infections were detected in 43/179 (24.02%) episodes, of which 37/179 (20.67%) were with two viruses, 5/179 (2.79%) were with three viruses, and 1/179 (0.55%) was with four viruses. The most common combination of two-virus coinfections was IFVA/AdV, followed by IFVB/Adv, IFVB/IFVA, RSV/IFVA, HMPV/AdV, RSV/AdV, and HMPV/IFVA. The result is shown in Table 3.

The combination of IFA and AdV were predominant among cases with three and four coinfecting viruses. Coinfection was more common in males 28/43 (65.11%) than females 15/43 (34.88%). Clinical data were available for 178 children that were infected with at least one virus, and the most common symptoms were fever and cough. There was no significant correlation between the clinical symptoms and the number of detected viruses.

## 4. Discussion

ARTI is one of the most important public health problems due to its high incidence and ease of spread in the society [8]. Respiratory viruses are the most common pathogens in the development of ARTI [4, 8]. The study of respiratory virus prevalence is important in the control and treatment of these infections [4]. RSV, IFVA/B, PIV1, 2, and 3, HMPV, and AdV are considered to be the most common causative viruses for ARTI [3]. We, therefore, evaluated the prevalence of IFVA, IFVB, RSV, HMPV, and AdV in children hospitalized with ARTI.

In our study, samples were obtained from symptomatic patients, and at least one respiratory virus was detected in 179/340 (52.64%) children. Perhaps, one of the reasons for the high prevalence of viral infections in this study is related to the study population because these patients had acute respiratory symptoms and were admitted to the hospital after initial treatment with the opinion of a specialist. In the study of Lei et al. [22], they found that the detection rate of viral respiratory infection among children hospitalized for ARI was 77.2%. Previous studies showed that about 31.2–86% of respiratory infection was caused by virus [23–25]. Bacteria or other respiratory viruses such as enteroviruses, coronaviruses, bocaviruses, or unidentified respiratory pathogens may be a cause of these undiagnosed infections [23]. Previous studies from Tabriz and Tehran reported that the prevalence of viral agents in samples from children with ARTI were (36%) [26] and (35.4%), respectively [27]. Studies in other countries have also shown that the prevalence of viral infections varies from 27% to 91.6% [28–38]. The large differences in viral detection rates may be due to heterogeneity within the study population, variability of genetic factors, the number and type of viral pathogens included for testing, methods used for testing, and geographic variation [34, 35, 39, 40].

In this study, the prevalence of viral respiratory infections was higher in boys than in girls. In general, men at younger and older ages are more susceptible to severe consequences of respiratory viral infections. This is despite the fact that during the reproductive years, women are often at greater risk than men with more severe consequences of viral respiratory infections. Maturity and gender influence the pathogenesis of respiratory viral infections [41].

In our study, the prevalence of viral respiratory infections in children under 5 years of age is higher than other ages. Most often, viral infections of the respiratory tract spread when children's hands come into contact with the nasal secretions of an infected person [42]. Children under 5 years of age are usually more exposed to viral respiratory infections due to the lack of knowledge about social healthcare.

In the present study, the prevalence of IFVA, AdV, IFVB, RSV, and HMPV infections was 28.23%, 24.70%, 8.52%, 3.23%, and 2.64%, respectively. Previous studies reported the detection rates for IFVA, IFVB, RSV, and AdV as 4.7%–46%, 2.5%–40%, 9.7%–28.0%, and 4%–16.9%, respectively [26].

The prevalence of respiratory viruses tends to increase in autumn and winter in temperate climates. High rates of transmission/infection in children and infants may be related to immature immune systems, lack of prior exposure to these pathogens, living conditions which promote overcrowding or environmental air pollution, and greater pathogen exposure. Lack of hygiene may also lead to a higher rate of infections in infants and young children [35, 43–45].

Although in this study, we showed significant differences in clinical data and symptoms between the different respiratory viruses studied (Table 2), a specific clinically recognizable pattern for each virus group cannot be defined because all respiratory viruses in terms of clinical symptoms overlap.

Clinicians frequently do not consider more than one aetiologic agent responsible for the respiratory infection and often order diagnostic tests for single pathogen, especially influenza viruses. However, in some patients with respiratory illness, coinfection with different viruses is common [46] Hence, we evaluated viral respiratory coinfection in pediatric patients. At the host level, the result of dual infection is usually viral interference, and in this case, the replication of one virus causes competitive inhibition of the other virus, but in some cases, it can increase viral replication. In coinfection, the time interval between virus exposure and the route of infection seems to affect the pathogenicity of coinfection [47]. Coinfections can also change the epidemiology of viral infections. For example, a fast-replicating virus can interfere with the reproduction of other viruses and inhibit the presence of other low-replicating viruses. The pathophysiology behind dual viral infections can explain some of the epidemiology of viral-viral infections seen at the population level [47].

In our study, multiple viral infections occurred in 24.40% (43/179) patients. In the study of Chun-Yu Yen et al., 27% of the children had multiple viral infections [48]. The result is similar to the previous studies that the detection of multiple viruses simultaneously in pediatric patients ranges from 10 to 30% [49].

In the present study, IFVA was the pathogen most commonly associated with viral-viral coinfections in ARTI patients. Although no statistically significant association was found between coinfecting viruses, Greer et al. [50] reported a negative associations between infection with human rhinovirus and AdV, HMPV, and RSV. Similarly, Brunstein et al. [51] reported a low prevalence of IFVA in the face of RSV, parainfluenza, HMPV, and human rhinovirus, albeit not statistically significant. Also, in a study by Tanner et al. [23], prominent associations between coinfecting respiratory viruses were reported with a lower frequency of AdV, RSV, parainfluenza, HMPV, and human rhinovirus in cases that were positive for IFVA and vice versa.

## 5. Conclusion

ARTI is a complex and diverse group of diseases that commonly affect infants and children and range in severity from mild to severe and is life threatening. Reliance on clinical symptoms to identify the causative pathogen is not possible, given the broad range of potential organisms. We describe a high prevalence of respiratory viral infections among Iranian children hospitalized with ARTI and a relatively high rate of viral-viral coinfections. IFVA was the most commonly detected virus, either monoinfection or coinfection.

There is a growing appreciation that viruses are involved in the pathogenesis of ARTI. The use of vaccines, including seasonal influenza vaccine, may be helpful in preventing ARTI in children and adolescents.

### 5.1. Limitation

In this study, the relationship between the viral load and clinical features was not defined. More research is needed to understand the prevalence of these viruses and their impact on disease severity in children with acute respiratory infections.

## Figures and Tables

**Table 1:** Primer sequences, product size, and annealing temperature.

Virus	Primer sequence	Product size (bp)	Method	Tm	Reference
AdV	TTCCCCATGGCICAYAACAC	482	Universal PCR	60°C	[17]
	CCCTGGTAKCCRATRTTGTA				

RSV	AACAGTTTAACATTACCAAGTGA	379	Universal PCR	58°C	[18]
	TGATTACTTGAGATATTGATGC				

HMPV	GAGCAAATTGAAAATCCCAGACA	347	Universal PCR	58°C	[19]
	GAAAACTGCCGCACAACATTTAG				

IFAV	GACCRATCCTGTCACCTCTGAC	106	One step RT-PCR	58°C	[20]
	AGGGCATTYTGGACAAAKCGTCTA				
	FAM-TGCAGTCCTCGCTCACTGGGCAGC- BHQ-1				

IFBV	TTCTTTCCCACCGAACCAAC	95	One step RT-PCR	58°C	[21]
	GAGACACAATTGCCTACCTGCTT				
	FAM-AGAAGATGGAGAAGGCAAAGCAGAACTAGC-BHQ-1				

**Table 2:** Demographic data of all the children admitted with acute respiratory infections.

	Respiratory infection (*n* = 340)	Viral respiratory infection (*n* = 179)	HMPV (*n* = 9)	RSV (*n* = 11)	INFB (*n* = 29)	INFA (*n* = 96)	Ade (*n* = 84)
*Sex*							
Male	208 (61.20%)	111 (62.01%)	7 (77.77%)	6 (54.54%)	19 (65.51%)	59 (61.45%)	53 (63.09%)
Female	132 (38.80%)	68 (37.98%)	2 (22.22%)	5 (45.45%)	10 (34.48%)	37 (38.54%)	31 (36.90%)

*Age*							
Min	1	1	1	1	1	1	1
Maxim	15	15	12	5	9	15	10
Mean ± sd	3.13 ± 2.38	3.30 ± 2.46	3.88 ± 4.04	2.36 ± 1.50	2.82 ± 1.89	3.40 ± 2.71	3.19 ± 2.23

*Age groups*							
<3	151 (44.41%)	75 (41.89%)	5 (55.55%)	6 (54.54%)	13 (44.82%)	42 (43.75%)	38 (45.23%)
3–5	108 (31.76%)	60 (33.51%)	1 (11.11%)	4 (36.36%)	10 (34.48%)	29 (30.20%)	26 (30.95%)
5–10	75 (22.06%)	40 (22.34%)	2 (22.22%)	1 (9.09%)	6 (20.68%)	22 (22.91%)	20 (23.80%)
10–19	6 (1.76%)	4 (2.23%)	1 (11.11%)	0	0	3 (3.12%)	0

*History*							
Underlying disease	16 (4.70%)	11 (6.14%)	0	0	2 (6.89%)	5 (5.20%)	6 (7.14%)
Travel	37 (10.88%)	16 (8.93%)	0	2 (18.18%)	5 (17.24%)	9 (9.37%)	8 (9.52%)
Influenza vaccination	16 (4.70%)	7 (3.91%)	1 (11.11%)	1 (9.09%)	0	5 (5.20%)	2 (2.38%)
Pneumonia	17 (5.00%)	9 (5.02%)	1 (11.11%)	1 (9.09%)	1 (3.44%)	3 (3.12%)	4 (4.76%)

*Symptoms*							
Fever	307 (90.29%)	164 (91.62%)	9 (100%)	11 (100%)	26 (89.65%)	89 (92.70%)	76 (90.47%)
Anorexia	145 (42.64%)	78 (43.57%)	1 (11.11%)	5 (45.45%)	19 (65.51%)	44 (45.83%)	30 (35.71%)
Runny nose	151 (44.41%)	73 (40.78%)	2 (22.22%)	5 (45.45%)	18 (62.06%)	39 (40.62%)	32 (38.09%)
Muscle pain	183 (53.82%)	94 (52.51%)	4 (44.44%)	7 (63.63%)	13 (44.82%)	47 (48.95%)	50 (59.52%)
Sore throat	175 (51.47%)	106 (59.21%)	5 (55.55%)	7 (63.63%)	15 (51.72%)	59 (61.45%)	46 (54.76%)
Headache	78 (22.94%)	41 (22.90%)	1 (11.11%)	1 (9.09%)	8 (27.58%)	25 (26.04%)	15 (17.85%)
Diarrhea	18 (5.29%)	11 (6.14%)	0	1 (9.09%)	2 (6.89%)	7 (7.29%)	4 (4.76%)
Cough	222 (65.29%)	116 (64.80%)	3 (33.33%)	9 (81.81%)	18 (62.06%)	62 (64.58%)	56 (66.66%)
Wheezing	176 (51.76%)	93 (51.95%)	5 (55.55%)	5 (45.45%)	14 (48.27%)	51 (53.12%)	46 (54.76%)
Bronchitis	26 (7.64%)	15 (8.37%)	1 (11.11%)	0	2 (6.89%)	7 (7.29%)	7 (8.33%)
Vomit	15 (4.41%)	8 (4.46%)	0	1 (9.09%)	2 (6.89%)	4 (4.16%)	3 (3.57%)

**Table 3:** Frequency of mono- and coinfection with respiratory viruses with a pattern.

			Frequency (*n* = 179)	Percent in each groups (%)
Monoinfection (*n* = 136) (53 female and 83 male)		HMPV	4 (2.2%)	2.94
		RSV	4 (2.2%)	2.94
		INFB	14 (7.8%)	10.29
		INFA	63 (35.2%)	46.32
		AdV	51 (28.5%)	37.5

Coinfection (*n* = 43) (15 female and 28 male)	Two viruses (*n* = 37)	HMPV, INFA	2 (1.1%)	5.40
		HMPV, AdV	2 (1.1%)	5.40
		RSV, INFA	3 (1.7%)	8.10
		RSV, AdV	2 (1.1%)	5.40
		INFB, INFA	5 (2.8%)	13.51
		INFB, AdV	6 (3.4%)	16.21
		INFA, AdV	17 (9.5%)	45.94
	Three viruses (*n* = 5)	RSV, INFA, AdV	2 (1.1%)	40
		INFB, INFA, AdV	3 (1.7%)	60
	Four viruses (*n* = 1)	HMPV, INFB, INFA, AdV	1 (0.6%)	100

		Total	179 (100%)	

## Data Availability

The data used in this study will be shared on request to the corresponding author with the permission of Mohammad Rahim Kadivar.
